# Nature of the lunar far-side samples returned by the Chang'E-6 mission

**DOI:** 10.1093/nsr/nwae328

**Published:** 2024-09-16

**Authors:** Chunlai Li, Hao Hu, Meng-Fei Yang, Jianjun Liu, Qin Zhou, Xin Ren, Bin Liu, Dawei Liu, Xingguo Zeng, Wei Zuo, Guangliang Zhang, Hongbo Zhang, Saihong Yang, Qiong Wang, Xiangjin Deng, Xingye Gao, Yan Su, Weibin Wen, Ziyuan Ouyang

**Affiliations:** Key Laboratory of Lunar and Deep Space Exploration, National Astronomical Observatories, Chinese Academy of Sciences, Beijing 100101, China; Lunar Exploration and Space Engineering Center, Beijing 100190, China; Beijing Institute of Spacecraft System Engineering, Beijing 100094, China; Key Laboratory of Lunar and Deep Space Exploration, National Astronomical Observatories, Chinese Academy of Sciences, Beijing 100101, China; Key Laboratory of Lunar and Deep Space Exploration, National Astronomical Observatories, Chinese Academy of Sciences, Beijing 100101, China; Key Laboratory of Lunar and Deep Space Exploration, National Astronomical Observatories, Chinese Academy of Sciences, Beijing 100101, China; Key Laboratory of Lunar and Deep Space Exploration, National Astronomical Observatories, Chinese Academy of Sciences, Beijing 100101, China; Key Laboratory of Lunar and Deep Space Exploration, National Astronomical Observatories, Chinese Academy of Sciences, Beijing 100101, China; Key Laboratory of Lunar and Deep Space Exploration, National Astronomical Observatories, Chinese Academy of Sciences, Beijing 100101, China; Key Laboratory of Lunar and Deep Space Exploration, National Astronomical Observatories, Chinese Academy of Sciences, Beijing 100101, China; Key Laboratory of Lunar and Deep Space Exploration, National Astronomical Observatories, Chinese Academy of Sciences, Beijing 100101, China; Key Laboratory of Lunar and Deep Space Exploration, National Astronomical Observatories, Chinese Academy of Sciences, Beijing 100101, China; Key Laboratory of Lunar and Deep Space Exploration, National Astronomical Observatories, Chinese Academy of Sciences, Beijing 100101, China; Lunar Exploration and Space Engineering Center, Beijing 100190, China; Beijing Institute of Spacecraft System Engineering, Beijing 100094, China; Key Laboratory of Lunar and Deep Space Exploration, National Astronomical Observatories, Chinese Academy of Sciences, Beijing 100101, China; Key Laboratory of Lunar and Deep Space Exploration, National Astronomical Observatories, Chinese Academy of Sciences, Beijing 100101, China; Key Laboratory of Lunar and Deep Space Exploration, National Astronomical Observatories, Chinese Academy of Sciences, Beijing 100101, China; Key Laboratory of Lunar and Deep Space Exploration, National Astronomical Observatories, Chinese Academy of Sciences, Beijing 100101, China; Institute of Geochemistry, Chinese Academy of Sciences, Guiyang 550081, China

**Keywords:** Chang'E-6, lunar samples, physical properties, mineralogical composition, rock type, chemical composition

## Abstract

The Chang'E-6 (CE-6) mission successfully achieved return of the first samples from the far side of the Moon. The sampling site of CE-6 is located in the South Pole-Aitken (SPA) basin—the largest, deepest and oldest impact basin on the Moon. The 1935.3 g of CE-6 lunar samples exhibit distinct characteristics compared with previous lunar samples. This study analyses the physical, mineralogical, petrographic and geochemical properties of CE-6 lunar scooped samples. The CE-6 soil has a significantly lower bulk density (0.983 g/cm^3^) and true density (3.035 g/cm^3^) than the Chang'E-5 (CE-5) samples. The grain size of the CE-6 soil exhibits a bimodal distribution, indicating a mixture of different compositions. Mineralogically, the CE-6 soil consists of 32.6% plagioclase (anorthite and bytownite), 19.7% augite, 10% pigeonite and 3.6% orthopyroxene, and with low content of olivine (0.5%) but high content of amorphous glass (29.4%). Geochemically, the bulk composition of CE-6 soil is rich in Al_2_O_3_ (14%) and CaO (12%) but low in FeO (17%), and trace elements of CE-6 soil such as K (∼630 ppm), U (0.26 ppm), Th (0.92 ppm) and rare-earth elements are significantly lower than those of the lunar soils within the Procellarum KREEP Terrane. The local basalts are characterized by low-Ti (TiO_2_, 5.08%), low-Al (Al_2_O_3_ 9.85%) and low-K (∼830 ppm), features suggesting that the CE-6 soil is a mixture of local basalts and non-basaltic ejecta. The returned CE-6 sample contains diverse lithic fragments, including local mare basalt, breccia, agglutinate, glasses and leucocrate. These local mare basalts document the volcanic history of the lunar far side, while the non-basaltic fragments may offer critical insights into the lunar highland crust, SPA impact melts and potentially the deep lunar mantle, making these samples highly significant for scientific research.

## INTRODUCTION

Returned natural samples are essential to planetary science research, providing key laboratory data to link orbital remote sensing observations to actual surface ground truth. A total of 382.981 2 kg of lunar samples have been collected from six Apollo missions, three Luna missions and the Chang'E-5 (CE-5) mission [1,2]. These lunar samples have provided critical information on the formation and evolutionary history of the Moon, contributing to the development of hypotheses such as the Moon's giant impact into early Earth origin, the Lunar Magma Ocean and the Late Heavy Bombardment. Collectively, these previous studies have significantly advanced the discipline of planetary science [3].

The study of CE-5 lunar samples has revealed the youngest volcanic rock samples found on the Moon, indicating that magmatic activity persisted until 2 billion years ago (Ga) [4,5]. This discovery challenged the prevailing understanding from the Apollo missions, which suggested that lunar magmatism ceased ∼3 billion years ago. As a result, it is reshaping our knowledge of the Moon's evolutionary history.

**Figure 1.**
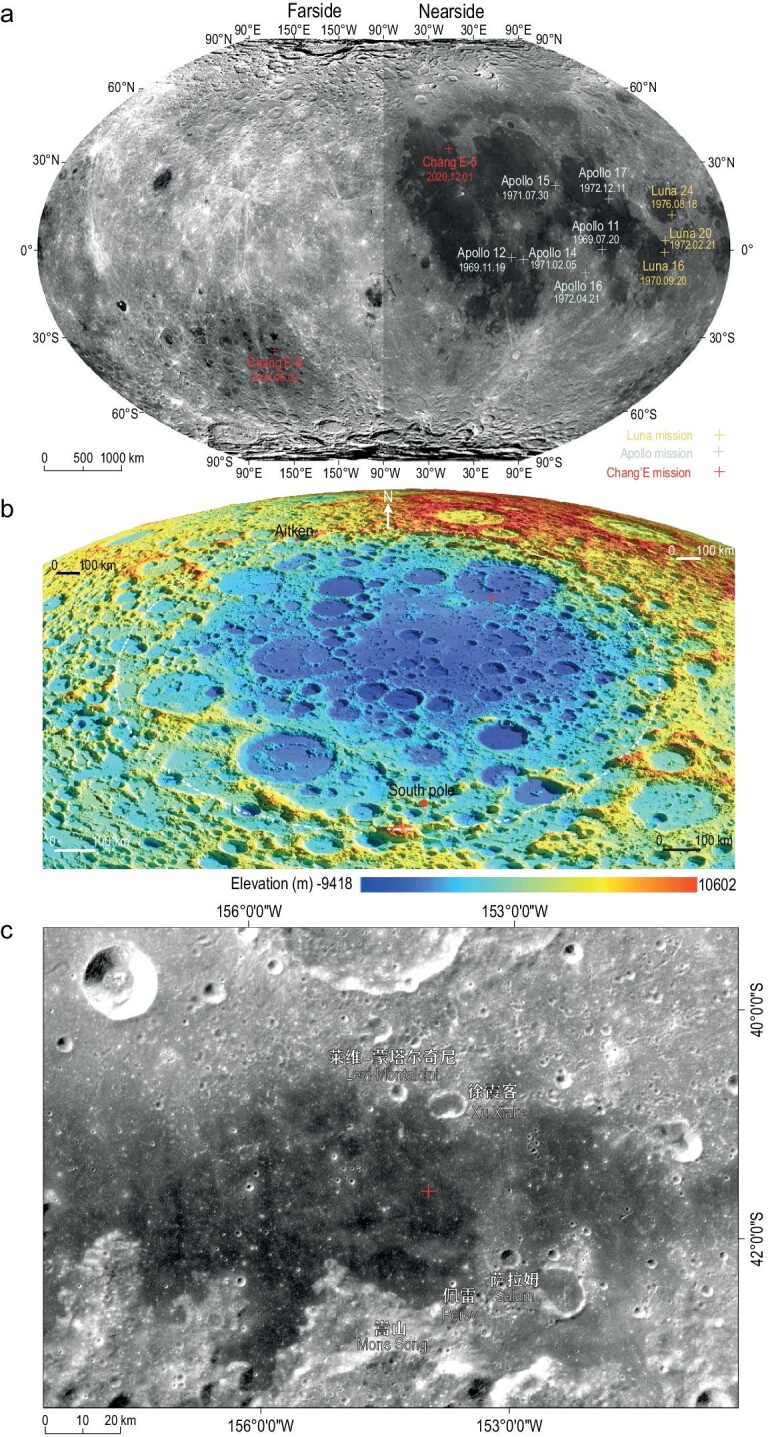
Map illustrating the distribution of lunar sampling sites and geological background of CE-6 sampling sites. (a) Distribution of lunar sampling sites and their collection dates. The sampling sites of Apollo, Luna and CE-5 missions are all situated on the nearside, with the exception of the CE-6 mission, which is located on the far side of the Moon. (b) Topographic map of the SPA region. (c) Image map of the CE-6 sampling point. The presence and distribution of the ejecta are more clearly shown in this enhanced image. The ‘+’ in both (b) and (c) mark the landing site of CE-6. The data used in (a) and (c) are derived from the global digital orthophoto map of CE-2 and the data in (b) are from the global digital elevation model of CE-1. All data are sourced from https://moon.bao.ac.cn.

However, all samples from the 10 Apollo, Luna and CE-5 missions were collected from the lunar nearside (Fig. 1a), leaving the far side unexplored from a sample perspective. A significant dichotomy exists between the nearside and far side of the Moon [6,7]. Nearside samples alone, without adequate sampling from the entire lunar surface, especially from the far side, cannot fully capture the geologic diversity of the entire Moon [8]. This limitation hampers our understanding of the Moon's origin and evolution. To help resolve this problem, on 25 June 2024, the Chang'E-6 (CE-6) mission collected 1935.3 g of lunar samples from the South Pole-Aitken (SPA) basin (41.625°S, 153.978°W) using scooping and drilling techniques. This represents the first time in history that lunar samples have been retrieved from the Moon's far side, ushering in a new and potentially revolutionary era of lunar scientific research.

This paper focuses on the preliminary investigation into the basic physical properties, rock types, petrography, mineralogy and geochemistry of the scooped samples obtained by the CE-6 mission, so as to provide foundational data for future in-depth scientific research to be carried out on these newly returned far-side samples.

## GEOLOGICAL CONTEXT

The lunar surface can be divided into three very distinct geochemical provinces based on variations in geochemical characterization and petrologic evolutionary history: the Procellarum KREEP Terrane (PKT), the Feldspathic Highland Terrane (FHT) and the South Pole-Aitken Terrane (SPAT) [7]. To date, lunar samples from the nearside, obtained by the Apollo, Luna and CE-5 missions, cover most of the PKT and include a few samples from the FHT (Fig. 1a). However, no samples have yet been returned from the unique SPAT on the lunar far side. The SPA basin, thought to have been formed 4.2–4.3 Ga ago in the pre-Nectarian period [9,10], is the largest confirmed impact basin in the Solar System. The massive impact that created the ∼2500 km diameter SPA basin likely excavated deep lunar material that may remain in the primordial basin [11–14]. The Apollo basin, in which the CE-6 samples were collected, is peak-ring basin with a 490-km diameter and is the deepest and largest impact structure within the SPA basin (Fig. 1b). The gravity data indicate that this area has one of the thinnest crusts on the entire Moon, making it highly probable that mantle material was excavated during the impact event [15]. Therefore, the landing site of CE-6 holds great scientific value for studying the early impact history of the Moon, understanding the composition of the deeper material and exploring the asymmetry between the Moon's nearside and far side [6,7].

The CE-6 landing site is situated on a flat basalt plain between the peak-ring and the southern rim of the Apollo basin, surrounded by several elevated landforms such as kipukas and wrinkle ridges (Fig. 1c). Topographic analysis suggests that a west-to-east basalt overflow event occurred here [16,17]. Statistical dating of the impact crater indicates that the basalt strata near the landing site have an age of ∼2.79−2.87 Ga (Supplementary Note 1).

The dark basalt overflow plain at the CE-6 landing site is intersected by several superposed light-colored ejecta rays from different directions, with the most prominent rays coming from the northwest–southeast, although additional ejecta rays can be seen from the southwest–northeast. Analysis of the ejecta index [2], derived from the image map, shows that the sampling site is ‘contaminated’ by at least ∼13% of foreign materials compared with the darkest part of the sampling area (Fig. 1c). This suggests that the CE-6 samples contain >10% foreign materials, in addition to the local basalt [18]. FeO and TiO_2_ data obtained from mineral spectral analysis further confirm the presence of non-basaltic ejecta materials in the landing site [16,17].

## RESULTS

### Physical properties of CE-6 soil

**Figure 2.**
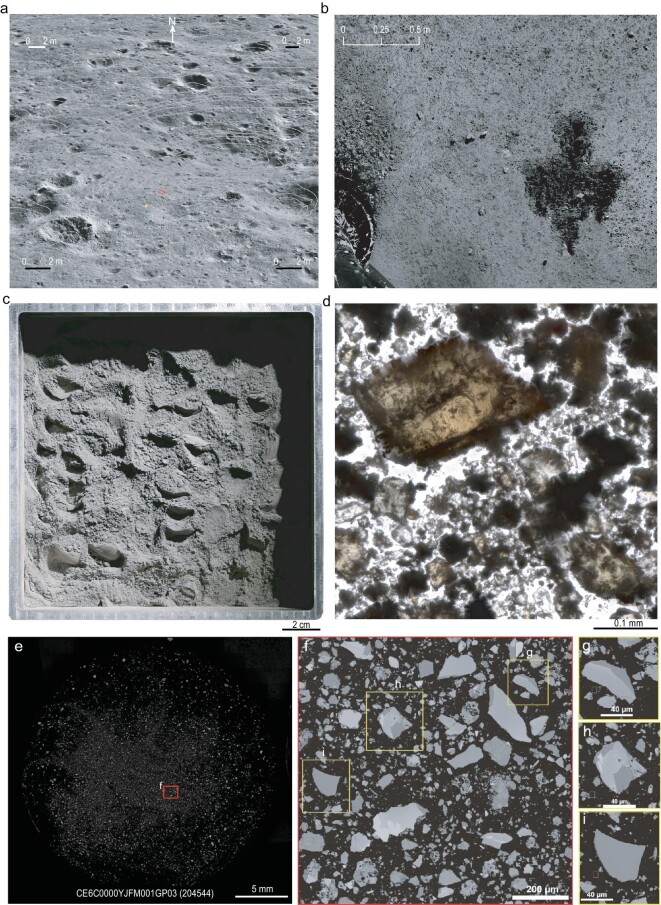
Image characteristics of CE-6 lunar samples. (a) A 3D map produced by CE-6 landing camera images at the sample collection site. The ‘+’ marks the central position of the lander, dots represent the drilling sample collection sites and polygon above the lander position indicate the scooping sample area. (b) Image of the scooped area on the lunar surface captured by the CE-6 panoramic camera. (c) Photo of the scooped lunar regolith obtained by using a laboratory camera. (d) Enlarged images of lunar soil particles under a stereomicroscope, showing particle-size distribution. (e) Backscattered electron (BSE) image of lunar soil. (f) An enlarged view of the area indicated in (e). (g–i) Typical BSE images representing single-mineral particle (I), dual-mineral particle (II) and three/more-mineral particle (III).

The CE-6 landing site is located at the flat edge of an impact crater with a diameter of ∼50 m and a depth of ∼3 m (Fig. 2a). CE-6 scooped samples consist of eight scoops from a single location, each with a scooping depth of ≤3 cm (Fig. 2b), yielding a total weight of ∼1610 g. Both on the lunar surface (Fig. 2b) and in Earth-based laboratories (Fig. 2c), the lunar regolith appears mostly gray–black. However, various clasts and mineral particles are easily identified under a stereomicroscope (Fig. 2d). Preliminary comparative observations indicate that the CE-6 soil contains significantly more light-colored particles (feldspar and glass) than the CE-5 soil, likely originating from impact ejecta rays or from depth from local impacts. The composition and structure of 382 264 CE-6 soil particles in three polished sections (CE6SC0QMJJ002GP, CE6SC0QMJJ005GP and CE6C0000YJFM001GP03) were counted and analysed by using backscattered electron (BSE) images (image resolution 0.28 μm) (Fig. 2e). The statistical results reveal that the average percentages of single-mineral particles, dual-mineral particles and three/more-mineral particles in lunar soil are 18.3%, 15.0% and 66.7%, respectively. By comparison, the CE-5 soil contains 27.0% single-mineral particles, 21.5% dual-mineral particles and 51.5% three/more-mineral particles [2]. These differences suggest a higher percentage of three/more minerals and a lower percentage of single minerals in the CE-6 soil compared with the CE-5 soil, which might reflect a lower degree of disaggregation and weathering of the original bedrock at the CE-6 landing site.

During the formation and accumulation of lunar soil, the primary factors that influence the physical properties are density, particle size, particle shape and space weathering [19]. Consequently, this study measured the physical properties of the CE-6 lunar soil, including the soil density and particle size.

#### Density of CE-6 lunar soil

We measured the bulk density of the CE-6 scooped soil sample (CE6C0000YJFM00101) in its natural state and determined the true density by using a Quantachrome ULTRAPYC 1200e analyser. The bulk density of the CE-6 scooped soil is 0.983 g/cm^3^, while the true density is 3.035 g/cm^3^. Both values fall within the density range measured for Apollo lunar soils (bulk density: 0.75−2.29 g/cm^3^; true density: 2.9−3.24 g/cm^3^) [19], but are significantly lower than the density of CE-5 lunar soils (bulk density: 1.2387 g/cm^3^; true density: 3.1952 g/cm^3^) [2]. This suggests the apparent influence of light components, such as feldspar and glass, and may also indicate a higher porosity for CE-6 lunar soils.

#### Particle-size distribution of CE-6 lunar soil

**Figure 3.**
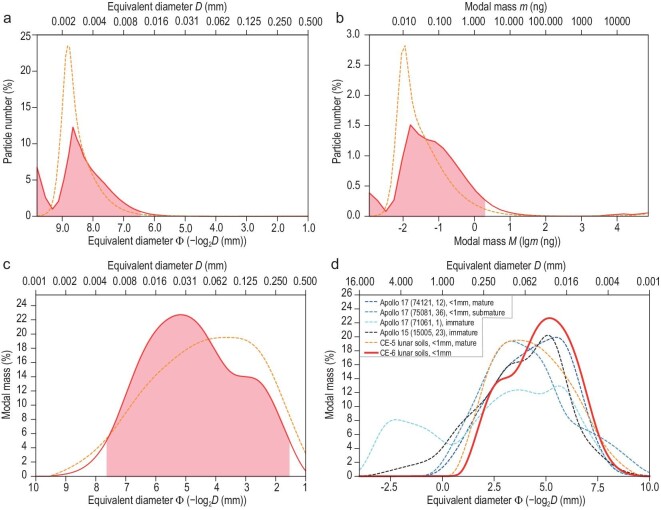
Particle-size distribution of CE-6 lunar soils. (a) The number (%) distribution of particle sizes (equivalent diameter). Particle sizes range from 1.11 to 529.25 μm, with a mean of 5.41 μm, a median of 7.91 μm and a mode of 2.49 μm. Of the particles, 95% (the red part) are distributed between 1.11 and 11.23 μm. (b) The modal mass (%) distribution of particle sizes. The modal mass ranges from 0.0011 to 123 137.5737 ng, with a mean of 0.8930 ng, a median of 0.0592 ng and a mode of 0.0160 ng. Of the particle mass, 95% (the red part) is distributed between 0.0011 and 1.8769 ng. (c) The modal mass-grain size distribution of CE-6 lunar soils. Of the particle mass, 95% (the red part) is distributed between 5.12 (Φ7.61) and 336.81 μm (Φ1.57), with a mean of 38.98 μm (Φ4.68), a mode of 27.97 μm (Φ5.16) and a median (*Φ*_50_) of 35.03 μm (Φ4.83). (d) Comparison of modal mass-grain size distribution among CE-6, CE-5 and Apollo lunar soils with varying degrees of maturity. CE-5 lunar soil data are from Ref. [2]; Apollo lunar soil data are from Ref. [26].

Particle-size distribution is a fundamental physical parameter of lunar soil, influencing the strength, compressibility, optical properties and thermal properties, and is also related to the evolution and accumulation rates of lunar soil [2,19]. In this study, 60 mg of soil (CE6C0000YJFM00104) was randomly selected from the CE-6 scooped sample. Well-dispersed lunar soil particles were analysed by using an optical microscope (image resolution 0.4 μm) and the particle-size data were obtained for 72 081 923 soil particles that were smaller than 1 mm. The results show that, quantitatively, 95% of the particles in the CE-6 scooped samples have a particle size (equivalent circular diameter *D*) distribution ranging from 1.11 to 11.23 μm (mean 5.41 μm, Fig. 3a), which belongs to the clay (<3.91 μm) to fine-silt (3.91−15.63 μm) class [20]. In terms of mass, 95% of the soil particles were distributed between 0.0011 and 1.8769 ng, with a mean value of only 0.8930 ng, a mode value of 0.0160 ng and a median value of 0.0592 ng (Fig. 3b). However, regarding the particle-size mass distribution [21], 95% of the mass of particles in the CE-6 soil had a particle-size distribution of between 5.12 (*Φ*7.61) and 336.81 μm (*Φ*1.57), with a mean value (= (*Φ*16 + *Φ*50 + *Φ*84)/3) of 38.98 μm (*Φ*4.68), a mode value of 27.97 μm (*Φ*5.16) and a median value (*Φ*50) of 35.03 μm (*Φ*4.83). The particle-size distribution exhibits a bimodal feature, with the majority of CE-6 soil particle sizes concentrated at ∼39 μm in mass (Fig. 3c and d, and Table S1).

### Petrographic characteristics of CE-6 lunar samples

**Figure 4.**
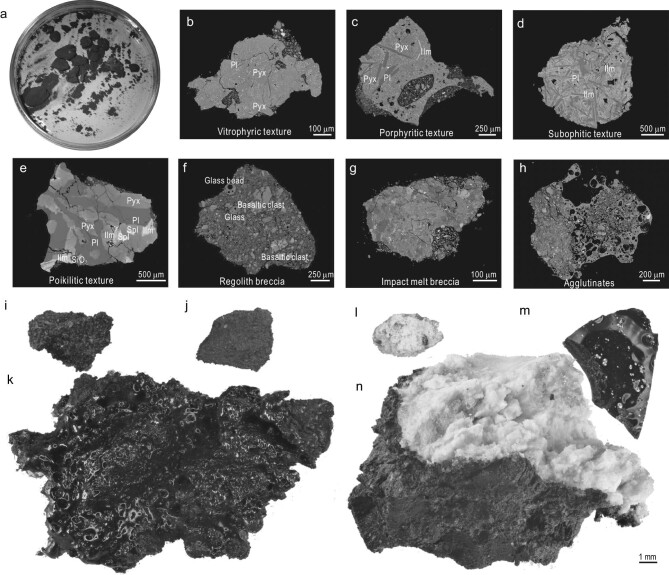
Typical images of lithic fragments in the CE-6 scooped sample. (a) Larger lithic fragments (>1 mm) picked from the CE-6 scooped samples. BSE image of (b–e) typical basaltic fragments with various textures, (f) regolith breccia, (g) impact melt breccia and (h) agglutinates. Stereomicrographs of (i) typical basalt, (j) regolith breccia, (k) agglutinate, (l) leucocratic and (m and n) glass fragments. Pyx, pyroxene; Pl, plagioclase; Ol, olivine; Ilm, ilmenite; Spl, spinel.

In addition to lunar soil, the CE-6 returned regolith samples that contained lithic fragments, typically larger than 1 mm (Fig. 4a). Based on rock-type analysis using stereomicroscopy and scanning electron microscopy, the lithic fragments could be classified into basalt, breccia, agglutinate, glass and leucocratic clasts.

#### Basalt

Basalt is the predominant rock type in the CE-6 samples, accounting for ∼30%–40% of the total lithic fragments collected. It is believed to be derived from the bedrock underlying the weathered lunar regolith at the sampling site. The mineralogy of CE-6 basalt is primarily composed of clinopyroxene, plagioclase and ilmenite, with very low olivine content. Basaltic fragments can be further divided into different textural subtypes based on their petrographic characteristics:

- Vitrophyric texture: the mineral grains are mainly composed of glass and extremely fine matrix minerals (needle-like microcrystals of plagioclase, pyroxene and ilmenite, typically <0.01 mm in size). The phenocrysts are predominantly clinopyroxene (grain size 0.05–0.1 mm), with a minor amount of slender plagioclase (Fig. 4b).
- Porphyritic texture: the phenocrysts are dominated by clinopyroxene and plagioclase with grain sizes of ≤0.25 mm, while olivine or spinel is rarely present (Fig. 4c). Matrix minerals are plagioclase, clinopyroxene and ilmenite, with fine grain-sizes of usually <0.05 mm. Plagioclase and ilmenite are acicularly oriented in the matrix, with clinopyroxene grains filling the plagioclase lattice.
- Subophitic texture: mineral grains are fine-grained (usually <0.1 mm), with euhedral slabs of plagioclase that form a lattice structure, which is filled with pyroxene grains. There are acicular or elongated ilmenite cuts through plagioclase and clinopyroxene (Fig. 4d and i).
- Poikilitic texture: plagioclase, pyroxene and ilmenite are coarse-grained (0.1–0.5 mm). They exhibit a complex intergrowth relationship, with plagioclase enclosing anhedral pyroxene grains. Ilmenite is relatively large and spinel occasionally appears (Fig. 4e and j).

In total, 55 basalt fragments of >1 mm were analysed during the petrological observation. The most common textures are poikilitic (48%) and subophitic (31%), followed by porphyritic (17%) and vitrophyric (∼4%).

#### Breccias

Breccia is another dominant lithic type in the CE-6 samples, comprising 30%−40% of the total lithic fragments. Based on their petrographic characteristics, breccias can be divided into regolith breccias and impact melt breccias (Fig. 4f and g). Regolith breccias are the predominant type, composed mainly of basalt clasts, glass beads and large irregular glass fragments, with occasional anorthositic and noritic material. In contrast, impact melt breccias are dominated by single-mineral fragments and leucocratic material (primarily anorthositic and noritic material), with occasional granulite clasts, and almost no basalt clasts. Unlike regolith breccias, impact melt breccias rarely contain glass beads or irregular glass fragments.

#### Agglutinates

Agglutinates are a special form of breccias, cemented by glassy matrix, and account for ∼20%−30% of the collected fragments. Most agglutinates are irregular in shape, loose and friable. Their surface is commonly covered by glass, which contains well-developed pores (Fig. 4h and j). The composition of agglutinates is complex, with mineral clasts consisting mainly of plagioclase, pyroxene, ilmenite and olivine, etc. The lithic clasts include basalt, impact melt breccia and leucocratic material. The glassy matrix often contains troilite and Fe–Ni metals.

#### Glassy materials

The glassy materials in CE-6 soils can be divided into two subgroups based on morphological differences. One subgroup consists of rounded glass beads that vary in color, including black, brownish-yellow, white and green. The second subgroup comprises irregular glass fragments (Fig. 4l and n), occasionally containing residual minerals. Both types of glassy material are mostly found in regolith breccias or as separate fragments.

#### Leucocratic fragments

In addition to occurring in breccias and agglutinates, leucocratic fragments (Fig. 4k) make up ∼10% of the collected lithic fragments. Preliminary petrographic analyses indicate that the lithology of leucocratic fragments is dominated by anorthosite and norite, with a very small amount of troctolite. The anorthosite consists mainly of An-rich plagioclase with minor pyroxene and olivine (<10 vol%). The norite is composed of plagioclase (48%, An = ∼96) and orthopyroxene (43%), with minor magnesium-rich olivine, chromite and troilite. The rare troctolitic fragment contains plagioclase and granular Mg-rich olivine, with some pyroxene occurring as interstitial minerals within the plagioclase grains.

### Mineralogy and geochemistry

#### Mineral species and abundance

The phase content analysis of CE-6 soil samples (CE6C0000YJFM00107, CE6C0000YJFM00108 and CE6C0000YJFM00109) was conducted using an X-ray diffraction analyser (XRD) (Supplementary  Note 2). The results indicate that the main phases are plagioclase (32.6%), augite (19.7%), pigeonite (10.0%) and amorphous glass (29.4%), with small amounts of orthopyroxene (3.6%), ilmenite (1.6%), olivine (0.5%) and other minerals (2.6%) (Table 1).

**Table 1.** Phases and abundances of CE-6 lunar soils.

	Samples and abundances (vol%)			
Phase	CE6C0000YJFM00107	CE6C0000YJFM00108	CE6C0000YJFM00109	Mean
Plagioclase	32.9	32.3	32.7	32.6
Augite	20.1	20.6	18.3	19.7
Pigeonite	10.2	10.5	9.3	10.0
Orthopyroxene	3.7	3.8	3.4	3.6
Ilmenite	1.8	1.5	1.4	1.6
Olivine	0.6	0.2	0.6	0.5
Amorphous glass	27.9	28.6	31.8	29.4
Others	2.8	2.5	2.5	2.6

The glass content of CE-6 soil is relatively low, approaching the lower limit of Apollo soils, which ranges from 25.4% to 72.3% [22,23]. The total pyroxene content in CE-6 soil reaches 33.3%, which is close to the upper limit found in Apollo soils (0.9%−33.8%). The plagioclase content (32.6%) is significantly higher than that of Apollo mare basalts (13.4%−20.0%) and even slightly higher than the lower range of plagioclase in Apollo 16 highland samples (28.1%−64.3%). The olivine content (0.4%) in CE-6 soil is on the lower end of the range found in Apollo soils (0.3%−4.8%). Compared with the CE-5 soil, the CE-6 soil contains a small amount of orthopyroxene, but the olivine content is notably lower.

#### Mineral composition of CE-6 soil

The composition of minerals, including plagioclase, pyroxene, ilmenite, ulvöspinel and olivine, from three lunar soil polished sections (CE6C0000YJFMGP01, CE6C0000YJFMGP02 and CE6C0000YJFMGP03) were analysed by using an electron probe microanalyser. All sections were prepared from the CE-6 scooped samples (Supplementary Note 3 and Table S2).

**Figure 5.**
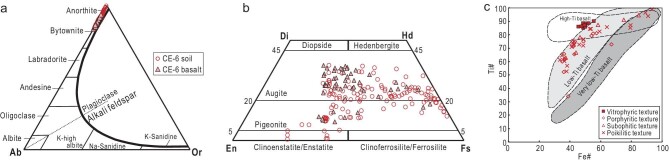
Mineral composition of (a) plagioclase and (b and c) pyroxene in lunar soils and basalt returned by the CE-6 mission. Plagioclase is primarily composed of anorthite and bytownite. Pyroxene is dominated by augite and pigeonite, with a minor amounts of enstatite. In the Fe# vs.Ti# plots, most pyroxenes from various basaltic textures fall within the low-Ti field.

The composition of plagioclase is relatively homogeneous, with An values ranging from 82.9 to 99.7 (*n* = 98, Fig. 5a). Nearly half of the plagioclase is anorthite, with an average composition of An_94.3_Ab_5.4_Or_0.3_ (*n* = 45), while the other half consists of bytownite, with an average composition of An_86.6_Ab_12.9_Or_0.6_ (*n* = 53). The pyroxene composition is variable, mostly composed of augite (average composition Wo_25.5_En_25.1_Fs_49.4_, *n* = 86) and pigeonite (average composition Wo_14.0_En_33.9_Fs_52.1_, *n* = 42), in addition to minor amounts of orthopyroxene (Wo_2.5_En_63.6_Fs_34.0_, *n* = 8) (Fig. 5b). The majority of olivine grains have Fo values of between 30 and 60. Of the remaining grains, 33% are Fe-rich (Fo < 15), 10% are Mg-rich (Fo > 75) and one olivine grain is extremely Mg-rich, with a Fo value of 93.

#### Mineral composition of CE-6 basalt

The major mineral compositions of basalt fragments with four different textures show that the anorthite content (An) in plagioclase varies from 84.0 to 93.6, with an average composition of An_88.8_Ab_10.9_Or_0.3_ (*n* = 37) (Fig. 5a). Pyroxene in the basalt is predominantly augite, with an average composition of Wo_29.6_En_34.4_Fs_36.0_ (*n* = 42) (Fig. 5b). Pigeonite is less abundant, with an average composition of Wo_12.7_En_40.7_Fs_46.6_ (*n* = 9). The composition of ilmenite is homogeneous, with average contents of 51.2% TiO_2_ and 46.4% FeO. According to the Fe# vs. Ti# correlation diagram of pyroxene, the basalts with porphyritic, subophitic and poikilitic textures in the CE-6 samples are all classified as low-Ti basalts (Fig. 5c). Although the composition of vitrophyric pyroxene falls within the range of the high-Ti basalts, petrographic evidence suggests that the ilmenite crystallized after the clinopyroxenes and plagioclase phenocrysts, which follows the typical crystallization sequence of low-Ti basalts [24]. Therefore, we conclude that the CE-6 basalts with different textures in this study are all low-Ti basalts.

#### Bulk chemical composition of CE-6 samples

**Figure 6.**
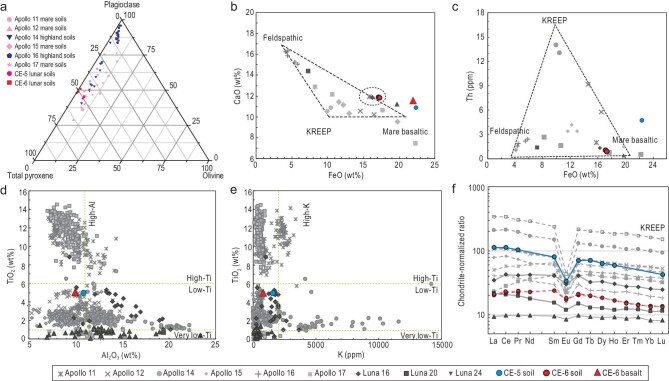
Mineralogy and geochemistry of CE-6 soils and basalt. (a) Triangular plot of major mineral abundances. CE-6 lunar soils are significantly enriched in pyroxene and low in olivine compared with CE-5. Data for Apollo and Luna soils are from Refs [22,24]. (b and c) Elemental variations of CaO, FeO and Th (database and triangles from Ref. [27]). (d and e) TiO_2_, Al_2_O_3_ and K classification scheme for mare basalts. The basaltic fragment from CE-6 belongs to the low-Ti/low-Al/low-K species (database from Ref. [28]). (f) Chondrite-normalized concentrations of rare-earth elements (REEs) in lunar soils as a function of an REE atom. The REE pattern of CE-6 lunar soils shows weak negative Eu anomalies. Database for Apollo samples is from Ref. [27] and KREEP composition data are from Ref. [29]. Normalization values: 1.36C, where C represents the ‘Mean C1 Chondr.’ values of Table 1 in Ref. [30].

The bulk chemical composition of CE-6 lunar soil and basalt was analysed by using X-ray fluorescence spectrometry (XRF) and inductively coupled plasma optical emission spectroscopy (ICP–OES) (Supplementary Notes 4 and 5). Additionally, the lunar soils CE6C0000YJFM00102 and CE6C0000YJFM00103 were further analysed for trace elements abundance by using laser ablation quadrupole inductively coupled plasma mass spectrometry (LA-Q–ICP–MS) (Table 2). Compared with samples from the Apollo and Luna missions, CE-6 lunar soils exhibit lower contents of CaO (∼12%) and Al_2_O_3_ (∼14%) and higher FeO (∼17%), which diverge from feldspathic and KREEP endmembers but are relatively close to the mare basalt end member (Fig. 6b and c). The bulk composition of CE-6 basalt fragments with subophitic texture (TiO_2_, ∼5%; Al_2_O_3_, ∼9.8%; and higher K_2_O, ∼0.1%) belongs to the low-Ti/low-Al/low-K species (Fig. 6d and e). The distinct compositional difference between soils and basalt from CE-6 samples indicates that exogenous material was incorporated during the regolith development of the basaltic bedrock.

**Table 2.** Chemical composition of CE-6 soils and basalt.

Element (wt%)	SiO_2_	TiO_2_	Al_2_O_3_	FeO	MnO	MgO	CaO	Na_2_O	K_2_O	P_2_O_5_	Total		Mg#		
CE6C0000YJFM00102 (soil)	45.6	2.7	14.3	17.3	0.22	7.08	11.9	0.25	0.07	0.07	99.42		42.2		
CE6C0000YJFM00103 (soil)	46.0	2.7	14.4	17.1	0.22	7.03	12.0	0.25	0.08	0.07	99.84		42.2		
CE6C0000YJYX41301^a^ (subophitic basalt)	45.0	5.08	9.85	22.0	0.28	5.19	11.5	0.38	0.1	0.09	99.52		29.8		
Element(μg/g)	Sc	V	Co	Ni	Ga	Rb	Sr	Y	Zr	Nb	Ba	La	Ce	Pr	Nd
CE6C0000YJFM00102 (soil)	40.7	71.6	41.6	268	5.91	2.11	180	32.6	117	8.26	79.0	6.47	18.1	2.77	14.0
CE6C0000YJFM00103 (soil)	41.4	63.1	33.3	210	5.83	2.19	175	32.2	119	7.93	72.9	6.75	18.7	2.84	14.0
Element(μg/g)	Sm	Eu	Gd	Tb	Dy	Ho	Er	Tm	Yb	Lu	Hf	Ta	Th	U	
CE6C0000YJFM00102 (soil)	4.80	1.31	5.66	0.99	6.08	1.27	3.29	0.47	3.05	0.44	3.61	0.33	0.90	0.26	
CE6C0000YJFM00103 (soil)	4.82	1.37	5.41	0.97	6.06	1.27	3.35	0.47	2.96	0.45	3.23	0.28	0.95	0.27	

a SiO_2_ content of CE6C0000YJYX41301 was calculated by subtraction of other major elements from 100 wt%.

The U, Th and K_2_O contents of CE-6 lunar soils are ∼0.27 ppm, ∼0.93 ppm and ∼0.08%, respectively. These values are significantly lower than those found in typical KREEP basalts, which contain U (4.0 ppm), Th (15.4 ppm) and K_2_O (0.5%) (Fig. 6c). The overall content of rare-earth elements (REEs) in CE-6 soils is only ∼20 times the chondrite normalized ratio (Fig. 6f), which is apparently lower than that of Apollo samples, but higher than that of Luna 20 and Luna 24 samples. The REE patterns for CE-6 lunar soils are relatively flat, with a weak negative Eu anomaly (δEu = Eu/Eu*, ∼0.8). Fractionation between light REEs and heavy REEs is not obvious. The overall REE pattern of CE-6 soil is similar to that of Luna 16 and Luna 20, while the concentration is approximately between these two soils.

## DISCUSSION

### CE-6 soil formation

The bulk density of the CE-6 soil is lower than that of most Apollo, Luna and CE-5 samples (1.1–2.1 g/cm^3^) [2,19], suggesting that the CE-6 lunar soil is looseer and more porous than previous lunar soils.

The true density of the CE-6 soil sample (3.035 g/cm^3^) is slightly lower than that of CE-5 soil (3.195 g/cm^3^) [2], which can be attributed to the higher abundance of low-density plagioclase and glass, and the lower abundance of olivine, consistently with the result of XRD measurement.

In comparison with CE-5 soil, the particle-size distribution of CE-6 soil exhibits a clear bimodal pattern. One peak (2.49 μm in number, 26.42 μm (Φ5.24) in modal mass) (Fig. 3a and c) is similar to that of the CE-5 mature soils, representing the mature component of the CE-6 soil, whereas the other peak may represent additional mixed fractions.

Approximately half of the Apollo 17 soil samples exhibit bimodal features, indicative of immature lunar soils, whereas submature and mature soils typically show single-peaked features [25]. Both the size-number and size-mass patterns of CE-6 soil (Fig. 3a and c) are distinctly bimodal and are similar to those of Apollo 15 and 17 immature lunar soils (Fig. 3d), suggesting that they may have formed through a mixing-dominated process [19,25]. Given the presence of fresh impact craters around the CE-6 landing site, the CE-6 soil may result from the mixing of mature lunar soil with freshly ejected materials. Its composition has the addition of exotic materials (e.g. distal ejecta) in addition to local basalts.

### Diversity of rock types in CE-6 lunar sample and their origins

Compared with the CE-5 sample, CE-6 soil contains significantly less olivine (0.5% in CE-6 vs. 4.3% in CE-5), a significantly higher content of amorphous glass (29.4% in CE-6 vs. 20.7% in CE-5) and a higher percentage of plagioclase (Fig. 6). Additionally, CE-6 soil contains a measurable amount of orthopyroxene, suggesting the presence of exotic non-basaltic ejecta. Based on the proportion of orthopyroxene in the total pyroxene content, it is estimated that the percentage of exotic ejecta in CE-6 soil is no less than 11%. This percentage could be even higher when considering the contribution of the plagioclase component.

Petrologically, the CE-5 sample consists predominantly of monotonous basalt, whereas the CE-6 samples contain significant amounts of exotic, non-basaltic rock fragments in addition to the local basalt. Both mineralogical and chemical evidence suggests an abundance of non-basaltic material in the CE-6 lunar soils. The high anorthite content (An > 95) probably originates from the anorthosite, while the presence of orthopyroxene may indicate noritic material.

In terms of major elements, the contents of Al_2_O_3_, CaO and FeO in the CE-6 soil lie along the mixing line between mare basalt and feldspathic material, which is consistent with the petrographic observation of leucocratic components in breccias. The FeO and Th variation diagram indicates that the composition of CE-6 soil remains close to the end member of mare basalt, while being distant from the KREEP end member, aligning with the distribution of major elements (Fig. 6c). Although the TiO_2_ content in the CE-6 basalt is 5.08%, the TiO_2_ in CE-6 soil (2.7%) is significantly lower than the ∼6.2 wt% that was detected by remote sensing in the landing area [17]. Additionally, the U, Th and K contents in CE-6 lunar soil are lower than those in typical KREEP basalts and all soils returned from PKT, but only slightly higher than those in Luna 20 and Luna 24 soil. This observation is also consistent with the major elements, which show a clear deviation from the KREEP (Fig. 6f).

## SUMMARY

The CE-6 mission returned the first samples from the far side of the Moon in the history of lunar exploration missions, marking a significant milestone in lunar exploration science and technical exploration capability. The returned sample reflects a mixture of ‘local’ basaltic material and ‘foreign’ non-mare material.

Based on our studies, the CE-6 samples collected from the lunar regolith can be broadly divided into fine-grained soils and lithic fragments (>1 mm). Due to long-term space weathering, the soil particles have reached dust-level scales, with few particles larger than 1 mm. In terms of number, 95% of the particles have sizes ranging from 1.11 to 10.77 μm, while, in terms of mass, 95% of the particles are distributed between 5.97 and 382.36 μm, with an average size of 85.86 μm. On Earth, the true density of the lunar sample is 3.035 g/cm^3^, while the natural bulk density is only ∼0.983 g/cm^3^, indicating that the lunar sample is quite loose and would be even fluffier in its ‘natural’ state on the lunar surface.

The rock fragments in the CE-6 samples are mainly basalt, breccia and agglutinates. The primary constituent minerals of the soils are plagioclase, pyroxene and ilmenite, with very low olivine abundance. The increased amount of plagioclase and the presence of orthopyroxene suggest that the CE-6 sample is a mixture of local basaltic material and anorthositic/noritic ejecta from adjacent regions.

Chemically, the CE-6 soil exhibits higher Al_2_O_3_ and CaO and lower FeO content compared with CE-5 basalt, suggesting that the CE-6 soil contains non-basalt ejecta in addition to low-Ti basaltic material. The significantly lower contents of U, Th, K and REEs in the CE-6 soil are consistent with major elements data, reinforcing the view that the CE-6 soil is primarily a mixture of local basalts and non-basaltic ejecta materials.

As the first lunar sample obtained from the far side of the Moon, the CE-6 sample will provide an unparalleled opportunity for lunar research. This unique sample will help to advance our understanding of several key aspects of lunar science, including (i) the Moon's early evolution; (ii) the variability of volcanic activities between the nearside and far side; (iii) the impact history of the inner solar system; (iv) the record of galactic activity record that is preserved in the lunar weathering layer; (v) the lunar magnetic field and its anomalies and duration; and (vi) the composition and structure of the lunar crust and mantle. These insights are expected to lead to new concepts and theories regarding the origin and evolution of the Moon, and refine its use as an interpretive paradigm for the evolution of the terrestrial planets.

## Supplementary Material

nwae328_Supplemental_File
