# Supplementary material for: Nature of the lunar far-side samples returned by the Chang'E-6 mission
Source: Natl Sci Rev. 2024 Sep 16;11(11):nwae328. doi: 10.1093/nsr/nwae328 (PMC11495410; doi:10.1093/nsr/nwae328)
Supplement: nwae328_Supplemental_File [file nwae328_supplemental_file.docx]

**Supplementary Data**

**Nature of the lunar farside samples returned by the Chang’E-6 mission**

Chunlai Li^1*^, Hao Hu^2*^, Meng-Fei Yang^3*^, Jianjun Liu^1^, Qin Zhou^1^, Xin Ren^1^, Bin Liu^1^, Dawei Liu^1^, Xingguo Zeng^1^, Wei Zuo^1^, Guangliang Zhang^1^, Hongbo Zhang^1^, Saihong Yang^1^, Qiong Wang^2^, Xiangjin Deng^3^, Xingye Gao^1^, Yan Su^1^, Weibin Wen^1^, Ziyuan Ouyang^1,4^.

1. Key Laboratory of Lunar and Deep Space Exploration, National Astronomical Observatories, Chinese Academy of Sciences, Beijing, 100101, China

2. Lunar Exploration and Space Engineering Center, Beijing 100190, China

3. Beijing Institute of Spacecraft System Engineering, Beijing 100094, China

4. Institute of Geochemistry, Chinese Academy of Sciences, Guiyang 550081, China

Contents

[Supplementary Notes 2](#_Toc176526036)

[Supplementary Note 1. Dating of the Chang’E-6 Landing Area 2](#_Toc176526037)

[Supplementary Note 2. XRD analysis of lunar soils 2](#_Toc176526038)

[Supplementary Note 3. EPMA analysis of the polished sections for lunar soil and basaltic fragment 3](#_Toc176526039)

[Supplementary Note 4. Major and trace elements analysis of lunar soil samples 3](#_Toc176526040)

[Supplementary Note 5. Major elements analysis of lunar basaltic fragment 3](#_Toc176526041)

[Supplementary Tables 5](#_Toc176526042)

[Supplementary Table 1. The particle shape and size statistical results 5](#_Toc176526043)

[Supplementary Table 2. The number of EMPA analysis points for the polished sections of lunar soil and basaltic fragment. 5](#_Toc176526044)

[Supplementary Table 3. N(1) values and age estimates for the Chang’E-6 landing site. 5](#_Toc176526045)

[Supplementary Table 4. Minerals used in the Rietveld whole-pattern fitting and the corresponding Powder Diffraction Files (PDF) of ICDD. 5](#_Toc176526046)

[Supplementary Figures 6](#_Toc176526047)

[Supplementary Figure 1. Boundary delineation map of the dating area for the Chang’E-6 landing site. 6](#_Toc176526048)

[Supplementary Figure 2. Crater Size-Frequency Distribution (CSFD) dating curve for the Chang’E-6 landing site. 7](#_Toc176526049)

[Supplementary Figure 3. The X-Ray diffraction patterns and Rietveld whole-pattern fitting results of the three lunar soil samples. 8](#_Toc176526050)

[Supplementary References 9](#_Toc176526051)

# Supplementary Notes

**Supplementary Note 1. Dating of the Chang’E-6 Landing Area**

To ensure the accuracy of the dating work, we first need to select areas with uniform texture as the focus of study. Based on the 7-meter high-resolution DOM image data from Chang’E-2, we initially delineated a region of dark mare basalt with relatively uniform reflectivity near the landing site (Supplementary Figure 1a). Subsequently, we combined the MI FeO distribution map [Lemelin et al., 2015] and LRO-WAC TiO_2_ distribution data [Sato et al., 2017], refining and confirming the boundaries of the region based on the principle of selecting areas with both high and uniformly distributed FeO and TiO_2_ contents (Supplementary Figure 1b and 1c). Ultimately, the final dating area was determined to cover 2769.63 square kilometers, with an average albedo (gray value) of 35.2 within the region.

Within the delineated dating area, we conducted detailed identification of craters and obtained crater frequency distribution data for the region. Due to the high density of small craters in the area, to avoid uncertainties introduced by the statistical analysis of small craters, we selected craters with diameters of D ≥ 300 meters for analysis. We then applied the NPF1983 [Neukum, 1984] and NPF2001 [Neukum et al., 2001] models for fitting. The results showed that the N(1) values were 2.36 × 10⁻³ km⁻² (NPF1983) and 2.42 × 10⁻³ km⁻² (NPF2001), corresponding to ages of 2.79 Ga and 2.87 Ga, respectively (Supplementary Table 3 and Supplementary Figure 2).

**Supplementary Note 2. XRD analysis of lunar soils**

Three lunar soils numbered CE6C0000YJFM00107, CE6C0000YJFM00108 and CE6C0000YJFM00109 were used for X-Ray Diffraction (XRD) analysis. The diffraction patterns of the three soil samples were collected using Bruker D8 Advanced X-ray diffraction instrument at NAOC, and the identification and quantification of the mineral phases were analyzed by the Rietveld whole-pattern fitting method [Bish and Post, 1993]. Sample preparation, XRD measurement process and conditions are the same to that of [Li et al., 2022].

The three CE-6 soil samples’ XRD diffraction patterns and the Rietveld full pattern fitting results by Jade software are shown in Supplementary Figure 3. The fitting errors (Weighted Residual Error, Rwp) of the three samples were less than 5.9%, 5.15%, 5.83% and 5.14%, respectively. The phases identified and involved in the whole pattern Rietveld fitting included plagioclase, augite, pigeonite, orthopyroxene, ilmenite, olivine, other minerals and the amorphous glasses. The standard diffraction patterns of each mineral phase were from the International Center for Diffraction Data (ICDD), and the corresponding card number for each mineral phase is listed in Supplementary Table 4.

## Supplementary Note 3. EPMA analysis of the polished sections for lunar soil and basaltic fragment

Major element compositions of plagioclase, pyroxene, ilmenite, ulvöspinel, and olivine were determined using an EPMA (JEOL JXA-8230) equipped with an energy disperse spectrometer (EDS, INCA) at NAOC. The minerals were analyzed at an accelerating voltage of 15 kV and an electron beam current of 10 nA with a spot diameter of 5μm. The peak counting time was 20 s for each element, and the background time was 10 s. Natural minerals and synthetic glasses were used as standards, and the detection limits for most elements were 0.01 wt.%–0.03 wt.%. All data were corrected for atomic number (Z), X-ray absorption (A), and fluorescence (F) effects [Reed, 1993]. The analyzed polished sections include lunar soil and basaltic fragment are listed in Supplementary Table 2.

**Supplementary Note 4. Major and trace elements analysis of lunar soil samples**

The XRF analysis of CE-6 lunar soil samples were conducted using a wavelength dispersion (WD) XRF (WD-XRF) spectrometer (PANalytical AXIOS Minerals) at the Institute of Geology and Geophysics, Chinese Academy of Sciences. The sample (30 mg) and lithium borate (3.0 g) were mixed thoroughly in the Pt–Au crucible at a ratio of 1:100. The sample was then melted at 1050℃ using an M4 propane gas automatic fluxer (Claisse, Quebec, Canada) and cast into a disk-shaped glass sample. The prepared disc-shaped glass sample was measured using WD-XRF (Malvern PANalytical, Almelo, Netherlands) [Xue et al., 2020]. Each glass disc was measured three times to calculate the average mass fraction of the major elements. Trace elements were subsequently analyzed using laser ablation quadrupole inductively coupled plasma mass spectrometry (LA-Q-ICP-MS) on lithium borate glass discs, employing an Agilent 8900 ICP-MS instrument (Agilent Technologies, Santa Clara, USA) coupled with a high-repetition-rate Genesis GEO Femtolaser Ablation System (Shanghai Chemlab Instrument Co., Ltd., Shanghai, China) [Eggins, 2003]. Five repeated measurements were conducted on different regions of each glass disc using linear laser ablation technology to calculate the average trace element mass fractions. Ablation was performed using spots with a diameter of 100 µm and a length of 1000 µm, at a frequency of 1 Hz for 45 s, following a 25 s measurement of the gas blank.

**Supplementary Note 5. Major elements analysis of lunar basaltic fragment**

The samples (CE6C0000YJYX41301, ~2 mg) were weighed and placed into Teflon bombs, where they were dissolved in a 0.5 ml mixture of concentrated HF and 50% HNO_3_ (1:1). The bombs were then securely inserted into stainless steel containers, which were sealed and heated at 190 °C in an electric oven for 16 hours. After cooling, the samples were evaporated to dryness. Subsequently, 0.1 ml of 50% HNO_3_ was added, and the samples were heated at 150°C in the electric oven for 1 hour until a second round of dryness was achieved. After cooling, a 2% v/v HNO_3_ solution was used to dilute the digested samples by a factor of 1500. The major element was determined using ICP-OES (IRIS Advantage, Thermo Fisher, USA). The instrument background was checked after each sample analysis. BHVO-2 and AGV-2 (USGS) standards were employed to monitor the preparation process, instrument performance, and the accuracy and precision of the analysis [Zong et al., 2022].

# Supplementary Tables

## Supplementary Table 1. The particle shape and size statistical results

| **Numbers** | **Statistical parameters** | **Minimum** | **Maximum** | **Median** | **Mode** | **Mean Size** |
| --- | --- | --- | --- | --- | --- | --- |
| 1 | Equivalent diameter^a^ (μm) | 1.11 | 529.25 | 7.91 | 2.49 | 5.41 |
| 2 | Model Mass^b^ (10^-6^mg) | 0.0011 | 123137.5737 | 0.0592 | 0.0160 | 0. 8930 |

**a.** The equivalent diameter (D) is defined as the diameter of a sphere or circle with the same projected area as the particle and is calculated as $D=2\sqrt{\frac{S}{\pi}}$. Here, S is the projected area which is the total number of pixels occupied by lunar soil particles multiplied by the square of the image resolution of the microscope, and the unit of S is μm^2^.

**b.** Model mass (m): an uncorrected mass of the lunar soil particles was first estimated from the equivalent sphere volume $V$ and the lunar soil density $\rho=3.0350 g/cm^{3}$. The equivalent sphere volume $V$ is the volume of a sphere calculated from the equivalent diameter of the particle, and the formula is $V=\frac{4}{3}\pi\left( \frac{D}{2} \right)^{3}$. In addition, a correction factor of $k=0.5227$ was derived based on the deviation between the actual weighed mass and the uncorrected mass of 11 lunar soil samples (10mg~20mg each). The final model mass (m) was calculated as $m=k\rho V$.

## Supplementary Table 2. The number of EMPA analysis points for the polished sections of lunar soil and basaltic fragment.

| **Sample numbers** | **Plagioclase**  **（points）** | **Pyroxene**  **（points）** | **Ilmenite**  **（points）** | **Ulvöspinel**  **（points）** | **Olivine**  **（points）** |
| --- | --- | --- | --- | --- | --- |
| **Lunar soil** | | | | | |
| CE6C00000YJFM001GP01 | 32 | 53 | 33 | 12 | 23 |
| CE6C00000YJFM001GP02 | 33 | 44 | 39 | ---- | 31 |
| CE6C00000YJFM001GP03 | 33 | 39 | 31 | ---- | 33 |
| **Basaltic fragments** | | | | | |
| CE6SC0QMJJ019GP03 | ---- | 9 | ---- | ---- | ---- |
| CE6C0000YJYX045GP01 | 27 | 35 | 30 | 4 | ---- |
| CE6C0000YJYX536GP01 | 10 | 7 | ---- | ---- | ---- |

## Supplementary Table 3. N(1) values and age estimates for the Chang’E-6 landing site.

| **Dating Model** | **Area (km²)** | **Crater Diameter Range (km)** | **N(1)[ km^-2^]** | **Age (Ga)** |
| --- | --- | --- | --- | --- |
| NPF1983 | 2769.63 | [0.30, 1.78] | 2.36× 10⁻³ | $2.79$ |
| NPF2001 |  |  | 2.42× 10⁻³ | $2.87$ |

## Supplementary Table 4. Minerals used in the Rietveld whole-pattern fitting and the corresponding Powder Diffraction Files (PDF) of ICDD.

|  | **Mineral** | **PDF numbers of ICDD** |
| --- | --- | --- |
|  | Plagioclase | PDF04-011-6816 |
|  | Augite | PDF98-000-0102 |
|  | Pigeonite | PDF01-076-2962 |
|  | Orthopyroxene | PDF04-013-2070 |
|  | Ilmenite | PDF04-012-1147 |
|  | Olivine | PDF01-075-8864 |
|  | Other minerals (Troilite and Quartz) | PDF04-009-7914 and PDF04-008-8461 |

# Supplementary Figures


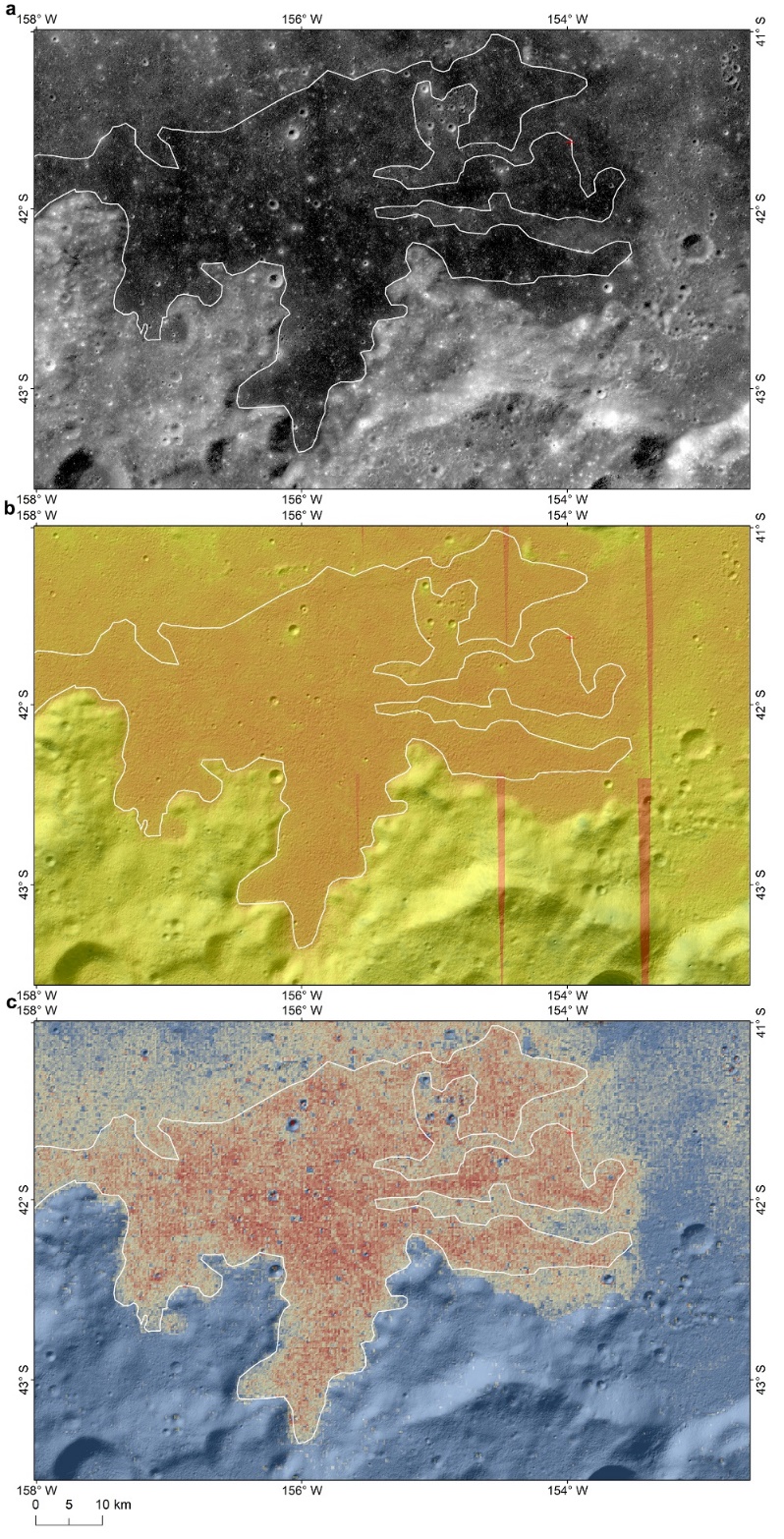


## Supplementary Figure 1. Boundary delineation map of the dating area for the Chang’E-6 landing site.

**a)** Area boundary delineated based on Chang’E-2 DOM image, **b)** Area boundary delineated based on the MI FeO distribution map, **c)** Area boundary delineated based on LRO-WAC TiO_2_ distribution data. the red cross marks the landing point of the Chang’e-6 spacecraft


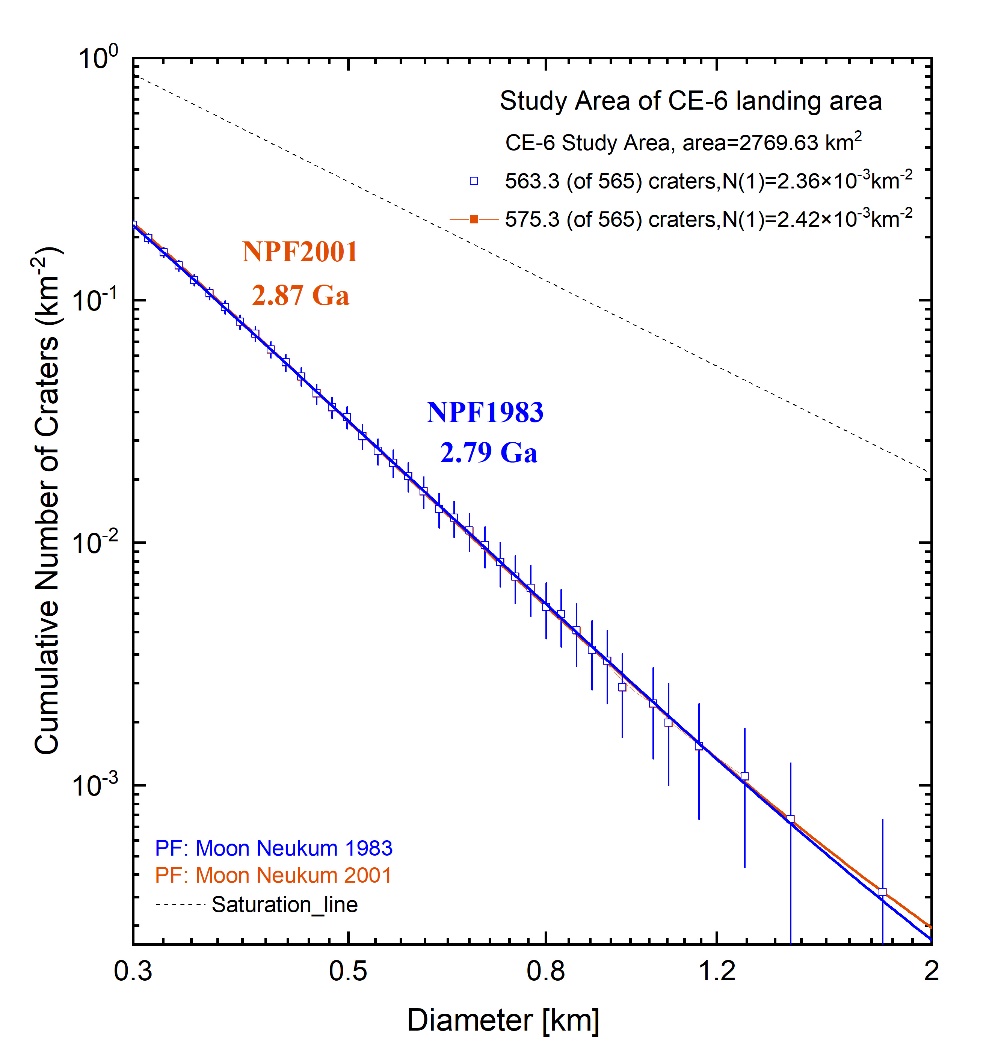


## Supplementary Figure 2. Crater Size-Frequency Distribution (CSFD) dating curve for the Chang’E-6 landing site.





## Supplementary Figure 3. The X-Ray diffraction patterns and Rietveld whole-pattern fitting results of the three lunar soil samples.

Original patterns and Rietveld Fitted Patterns represent XRD measured and fitted spectra of the samples, respectively. Difference Patterns show the difference between the fitted spectra and the measured spectra (offset 40000 for clarity). The difference pattern curves are all close to a straight line (40000), suggesting a good fitting result. Amorphous patterns are the diffraction spectra of the fitted glass. The backgrounds are the fitted background curves of the original patterns (offset 45000 for clarity).

# Supplementary References

1. Bish DL and Post JE. Quantitative mineralogical analysis using the Rietveld full-pattern fitting method. *Amer Min* 1993; **78**: 932-940.
2. Eggins SM. Laser Ablation ICP-MS Analysis of Geological Materials Prepared as Lithium Borate Glasses. *Geostand Newsl* 2003; **27**, 147–162.
3. Li CL, Hu H and Yang MF et al. Characteristics of the lunar samples returned by the Chang’E-5 mission. *Natl Sci Rev* 2022; **9**: nwab188.
4. Lemelin M, Lucey PG and Song E et al. Lunar central peak mineralogy and iron content using the Kaguya Multiband Image: Reassessment of the compositional structure of the lunar crust. *J Geophys Res Planets* 2015; **120**, 869–887.
5. Neukum G. Meteorite Bombardment and Dating of Planetary Surfaces; National Aeronautics and Space Administration: Washington, DC, USA, 1984.
6. Neukum G, Ivanov B and Hartmann WK. Cratering records in the Inner solar system in relation to the lunar reference system. *Space Sci Rev* 2001; **96**, 55–86.
7. Reed SJB. Electron microprobe analysis (2nd edition). Cambridge University Press, Cambridge, 1993.
8. Sato H, Robinson MS and Lawrence SJ et al. Lunar Mare TiO_2_ Abundances Estimated from UV/Vis Reflectance. *Icarus* 2017; **296**, 216–238.
9. Xue DS, Su BX and Zhang DP et al. Quantitative verification of 1:100 diluted fused glass beads for X-ray fluorescence analysis of geological specimens. *J Anal At Spectrom* 2020; **35**, 2826–2833.
10. Zong KQ, Wang ZC and Li JW et al. Bulk compositions of the Chang’E-5 lunar soil: Insights into chemical homogeneity, exotic addition, and origin of landing site basalts. *Geochim Cosmochim Acta* 2022; **335**, 284-296.
